# Osmium Nanoparticle Anchoring on CoFe Layered Double Hydroxides Enables High‐Valent Species Stabilization and Efficient Seawater Oxygen Evolution

**DOI:** 10.1002/advs.77998

**Published:** 2026-10-14

**Authors:** Xindong She, Guosheng Zhou, Kanghua Miao, Junjie Gong, Mingzhe Jiang, Xueting Zhu, Zhenzhen Wang, Shijie Shen, Wenwu Zhong

**Affiliations:** ^1^ Zhejiang Key Laboratory for Island Green Energy and New Materials School of Materials Science & Engineering Taizhou University Taizhou Zhejiang P. R. China; ^2^ Zhejiang Key Laboratory of Functional Ionic Membrane Materials and Technology for Hydrogen Production School of Chemistry and Chemical Engineering Shaoxing University Shaoxing Zhejiang P. R. China

**Keywords:** high‐valent metal sites, interfacial water structure, osmium nanoparticles, seawater oxygen evolution, selective adsorption

## Abstract

Direct seawater oxygen evolution reaction (OER) is critically hampered by chloride‐induced competitive oxidation, corrosion, and active‐site poisoning, driving the urgent need for highly active and chlorine‐tolerant electrocatalysts. Herein, a surface‐engineered electrocatalyst comprising osmium nanoparticles (Os NPs) anchored onto CoFe layered double hydroxide (Os/CoFe LDH) was prepared. X‐ray photoelectron spectroscopy revealed that the incorporated Os NPs stabilized high‐valence Co/Fe species, which preferentially promote OH^−^ adsorption over Cl^−^ and function as the genuine active centers for OER. Furthermore, in situ Raman spectroscopy demonstrates that Os NPs anchoring modulates the interfacial water structure, thereby enhancing water adsorption and facilitating seawater OER kinetics. The optimized Os/CoFe LDH‐40 achieves an overpotential of only 244 mV at 10 mA cm^−2^ in simulated alkaline seawater and operates stably for over 300 h in an anion exchange membrane electrolyzer at 300 mA cm^−2^. This work not only develops a high‐performance catalyst for alkaline seawater OER but also offers new perspectives for advancing osmium‐based materials in electrocatalysis.

## Graphical abstract

Osmium nanoparticles anchored on CoFe layered double hydroxide (Os/CoFe LDH) stabilize high‐valent Co/Fe species and modulate the interfacial water configuration via surface engineering. This work presents a facile surface engineering strategy for constructing efficient and durable OER electrocatalysts for alkaline seawater, and expands the applicability of Os‑based materials in electrocatalysis.

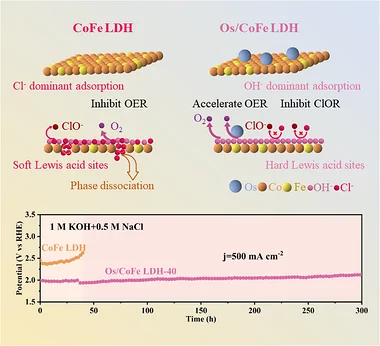

## 1 Introduction

Seawater electrolysis technology integrates seawater resources with renewable energy [1, 2, 3, 4], opening a new pathway for the large‐scale production of green hydrogen. In this technology, the anodic oxygen evolution reaction (OER) serves as the kinetic bottleneck [5, 6, 7] and directly constrains the overall energy conversion efficiency [8, 9]. However, the complex chemical composition of seawater causes multiple interferences with catalytic surface and interface reactions [10, 11, 12]. Notably, the abundant Cl^−^ in seawater participates in chloride oxidation reaction (ClOR) that occur at potentials similar to those required for the OER, thereby reducing the Faraday efficiency of oxygen production. Furthermore, depending on the pH of the reaction medium, ClOR generates various toxic and corrosive by‐products, such as Cl_2_, HClO, or ClO^−^, which severely shorten the service life of electrolyzer components. Thus, developing high‐performance, long‐lasting seawater OER catalysts is of great significance.

According to the hard‐soft acid‐base theory, catalysts exhibiting hard Lewis acidity preferentially coordinate with harder bases [13]. OH^−^ are harder Lewis bases compared to Cl^−^ and catalysts featuring high‐valence metal sites can manifest hard Lewis acidity, which renders the adsorption of Cl^−^ more thermodynamically unfavorable relative to OH^−^, thereby significantly prolonging the operational lifespan of the electrolysis system [14]. Besides, further augmenting catalytic activity to prevent the attainment of the ClOR potential that competes with the OER is equally critical. It is generally acknowledged that under anodic polarization conditions, high‐valence metal sites within catalysts always serve as the genuine active centers for the OER [15]. Accordingly, extensive efforts have been devoted to the deliberate construction of high‐valence metal sites to elevate catalytic activity. For instance, through fluorination engineering, the incorporation of fluorine atoms, possessing the highest electronegativity, into NiFe LDH enables the construction of electron‐deficient sites at Ni centers, elevating Ni intrinsic valence states [16]; via anion engineering strategies, sulfate‐induced NiFe LDH facilitates the generation of a higher proportion of high‐oxidation‐state metal species [17]; through metal doping, the modification of NiFe LDH with Ag and Ru dopants significantly augments the proportion of Ni^3+^ within the catalyst, consequently boosting OER activity [18].

Osmium (Os), characterized by a high electronegativity of 2.2, surpasses that of typical transition metals, endowing it with a more potent capacity to attract electrons. Upon the formation of a heterojunction interface between Os and other materials (e.g., LDH, metallic alloy), electrons always undergo spontaneous migration from the metal with lower electronegativity to Os. For example, Fu et al. [19] constructed an interstitial boron‐doped osmium, where theoretical and experimental analysis proved that the interstitial boron triggers electron enrichment at the Os site and the formation of negative charge centers. Ren et al. [20] prepared Os‐CoP/NF, and proposed that electron flow from Co to Os and consequently raising the Co oxidation state in Os‐CoP, which arose from Os exhibiting a higher electronegativity than Co (1.88). Consequently, research on leveraging cost‐effective Os (comparing to other platinum group metal) to stabilize the high‐valence states of non‐precious metals, thereby enabling efficient and stable seawater OER, holds significant scientific merit and application value.

Herein, we anchored Os NPs onto the surface of CoFe LDH to construct an Os/CoFe LDH catalyst, achieving concurrent high activity and durability through the stabilization of high‐valent Co and Fe species. X‐ray photoelectron spectroscopy (XPS) analysis confirmed that Co and Fe species remain high‐valent states after Os loading. In situ Raman spectroscopy verified that Os NPs loading modulates the interfacial water structure, diminishing the proportion of free water, while enhancing interfacial water adsorption, which is conducive to seawater OER. Electrochemical evaluations revealed that the optimized Os/CoFe LDH‐40 exhibits exceptional OER activity, requiring overpotentials of merely 244 mV to attain a current density of 10 mA cm^−2^ in simulated alkaline seawater. Moreover, an anion‐exchange membrane water electrolyzer (AEMWE) constructed with Os/CoFe LDH‐40 as the anode catalyst and Pt/C as the cathode catalyst, demonstrated excellent stability, sustaining continuous operation for over 300 h at 300 mA cm^−2^. This study establishes a straightforward surface engineering strategy for the fabrication of efficient and durable alkaline seawater OER catalysts, broadening the utility of Os‐based materials in the realm of electrocatalysis.

## 2 Results and Discussion

### 2.1 Synthesis and Characterizations

A two‐step synthesis method was developed to prepare Os‐based catalysts with tunable coordination structures on LDH. First, CoFe LDH was synthesized via co‐precipitation. Subsequently, Os NPs were anchored onto the CoFe LDH by introducing dilute solutions of OsCl_3_ and NaOH into a homogeneous LDH colloid, as illustrated in Figure 1a. To optimize the Os loading, a series of samples was prepared, including pristine CoFe LDH and Os/CoFe LDH‐x with different Os contents. The optimal catalyst, designated as Os/CoFe LDH‐40, was synthesized using 40 mg of OsCl_3_·xH_2_O. The Os content of Os/CoFe LDH‐40 was determined to be 5.94 wt.% by inductively coupled plasma‐mass spectrometry (ICP‐MS, Table S1). Morphological characterization was performed via scanning electron microscopy (SEM) and transmission electron microscopy (TEM). SEM images (Figure 1b and Figure S1) revealed that both CoFe LDH and Os/CoFe LDH‐x exhibited uniform nanosheet structures, with no significant morphological changes after Os loading. TEM images (Figure 1c) further showed Os NPs uniformly anchored on the nanosheets, exhibiting no significant aggregation. High‐resolution TEM (HRTEM) analysis (Figure 1d) showed distinct lattice fringes with an interplanar spacing of 2.3 Å, corresponding to the (110) plane of metallic Os, confirming the crystalline nature of the deposited Os NPs. The selected area electron diffraction (SAED) pattern displayed clear diffraction rings corresponding to the (110) and (101) planes of CoFe LDH, confirming its high crystallinity. Furthermore, high‐angle annular dark‐field scanning transmission electron microscopy (HAADF‐STEM) and the corresponding energy‐dispersive spectrometer (EDS) elemental mapping (Figure 1e) unequivocally demonstrated the uniform spatial distribution of Os, Co, Fe, and O throughout the Os/CoFe LDH‐40 nanosheets, and the size of the Os NPs is 4–5 nm. Fourier transform infrared (FTIR) spectroscopy provided insights into the chemical structure of the nanomaterials (Figure S2). Sharp peaks at approximately 3355–3448 cm^−1^ and 1630 cm^−1^ were attributed to the stretching vibrations of O─H groups in the interlayer space and water molecules within the crystalline structure, respectively [21]. The peak at approximately 1356 cm^−1^ originated from the stretching vibrations of NO_3_^−^ in the LDH interlayers [22]. Bands below 800 cm^−1^ were generally associated with lattice vibrations of metal‐oxygen bonds in LDHs (M─O stretching and M─OH bending), indicating the possible formation of Co─O, Fe─O, and Os─O bonds [23].

**FIGURE 1:**
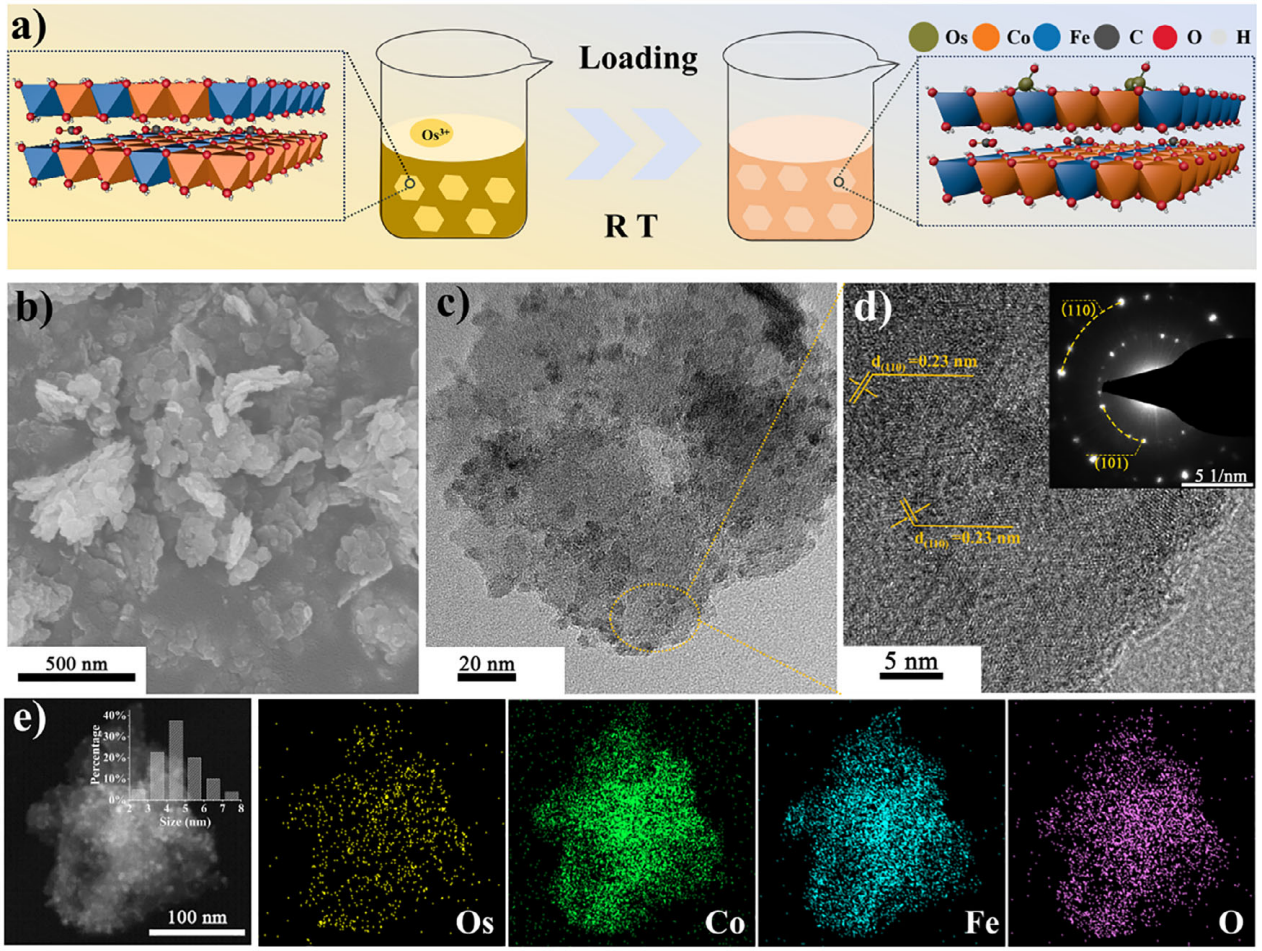
Preparation and morphology characterizations of Os/CoFe LDH. (a) Schematic illustration of the synthesis process of Os/CoFe LDH. (b) SEM image of Os/CoFe LDH‐40. (c) TEM image of Os/CoFe LDH‐40. (d) HRTEM image of Os/CoFe LDH‐40 with SAED pattern. (e) HAADF‐STEM image of Os/CoFe LDH‐40 (inset is the size distribution of Os NPs) along with corresponding Os, Co, Fe, and O element mappings.

The crystalline properties of the samples were characterized by powder X‐ray diffraction (XRD). Figure 2a displays the XRD patterns of the as‐prepared CoFe LDH and Os/CoFe LDH‐x, revealing sharp diffraction peaks at 11.6° and 23.4°, corresponding to the (003) and (006) planes of CoFe LDH [Inorganic Crystal Structure Database (ICSD) 500235], respectively [24], confirming the primary lattice structure of CoFe LDH. Importantly, the main lattice diffraction peaks did not shift after Os loading, as observed in the Os/CoFe LDH‐x, indicating that the fundamental LDH structure remained unchanged. This implied that Os species likely exist as NPs on the surface or in the interlayer region, rather than being incorporated into the lattice to substitute Co/Fe sites. To investigate the elemental composition and chemical states, crucial for understanding potential electronic interactions and catalytic activity, XPS analysis was performed. XPS survey spectra (Figure S3) revealed that, in addition to Co, Fe and O, Os is present in the catalysts after the Os loading process. The high‐resolution Co 2p XPS spectrum in Figure 2b consists of 2p_3/2_ and 2p_1/2_ core levels, involving Co^2+^ and Co^3+^ oxidation states [25]. The binding energies for these two oxidation states are 781.7 and 779.9 eV for the Co^2+^ 2p_3/2_ and Co^3+^ 2p_3/2_ level, and 797.1 and 795.8 eV for the Co^2+^ 2p_1/2_ and Co^3+^ 2p_1/2_ level, respectively. Similarly, the high‐resolution Fe 2p XPS spectrum revealed 2p_3/2_ and 2p_1/2_ core levels, corresponding to both Fe^2+^ and Fe^3+^ oxidation states [26]. Figure 2c shows the Fe 2p XPS spectrum, with binding energies of 711.1 eV (Fe^2+^ 2p_3/2_), 713.9 eV (Fe^3+^ 2p_3/2_), 723.9 eV (Fe^2+^ 2p_1/2_), and 726.1 eV (Fe^3+^ 2p_1/2_). Satellite peaks were observed at 718.6 and 733.5 eV. The high‐resolution Os 4f XPS spectra in Figure 2d were consistent with the Os 4f_7/2_ and 4f_5/2_ energy levels. The observed binding energies at 52.6 and 55.8 eV confirmed the presence of the Os^2+^ oxidation state in the 4f level, corresponding to Os loading into the LDH structure [27]. However, no peak corresponding to the Os 4f energy level was observed in Os/CoFe LDH‐5, which might be due to the extremely low loading amount of Os. In Figure 2e, the high‐resolution O 1s XPS spectra of CoFe LDH and Os/CoFe LDH‐x showed two peaks at 529.5 and 531.3 eV assigned to metal‐oxygen and metal‐hydroxide, respectively. A comparative analysis of the peak positions of Co and Fe in Os/CoFe LDH‐40 vs. those in pristine CoFe LDH revealed significant shifts in their binding energies. These shifts indicated strong electronic interactions between the loaded Os and the Co/Fe species [28]. Quantitative XPS analysis, considering the relative proportions of Co and Fe peaks, further elucidated these changes. Specifically, the ratio of Co^3+^ to Co^2+^ increased from 1:1.3 in CoFe LDH to 1:0.7 in Os/CoFe LDH‐40 (Figure 2f and Table S2), signifying an enhanced presence of Co^3+^ ions after Os loading. The Fe^3+^ to Fe^2+^ ratio changed from 1:1.1 in CoFe LDH to 1:1 in Os/CoFe LDH‐40, suggesting a relative increase in Fe^3+^ content. This redistribution of charge and oxidation states, particularly the increase in Co^3+^ and Fe^3+^, implies the retention of high‐valent Co and Fe states following Os loading. This electronic tuning is critical for optimizing the catalytic performance of the Os/CoFe LDH‐40.

**FIGURE 2:**
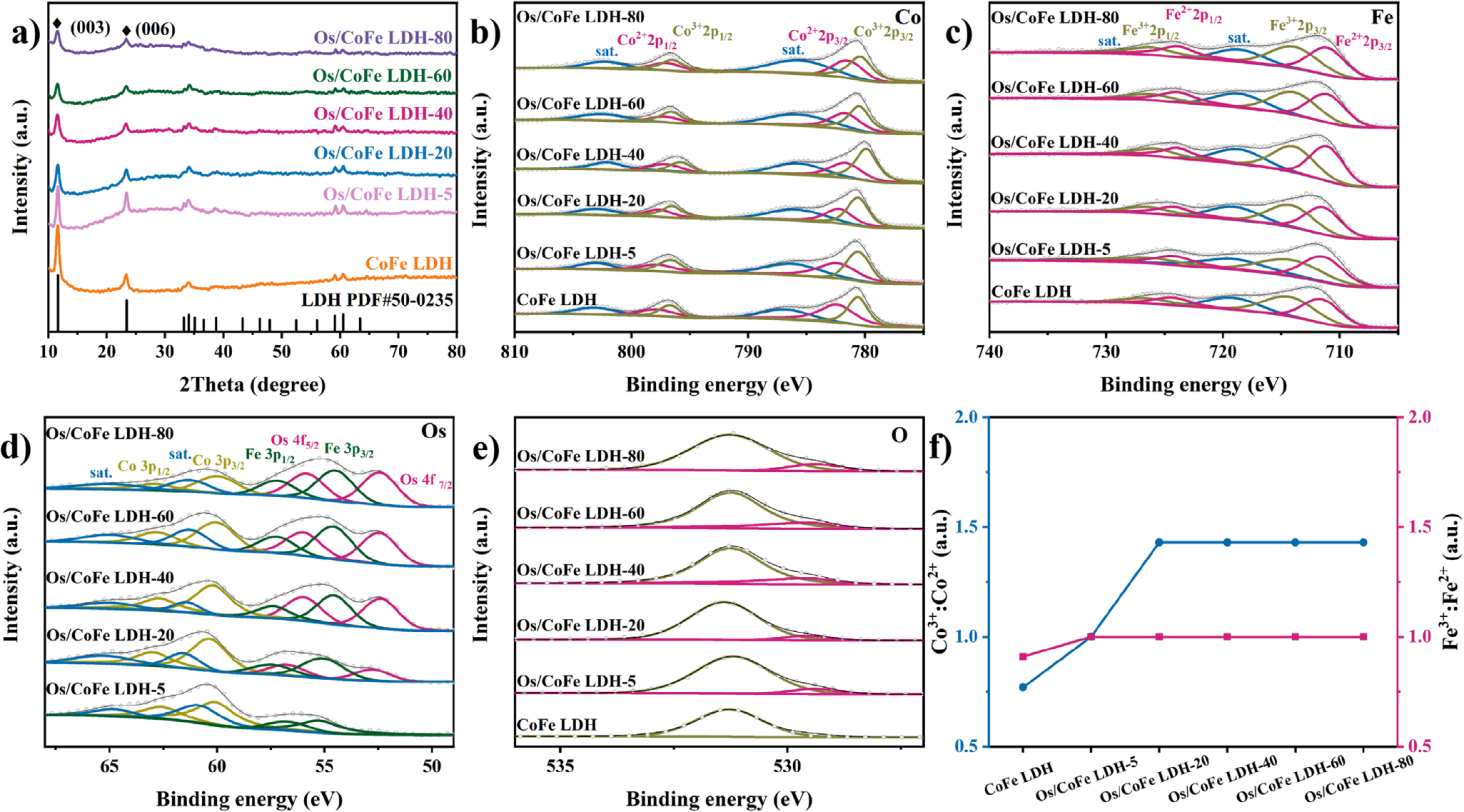
Structural characterizations of CoFe LDH and Os/CoFe LDH‐x. (a) XRD patterns. (b) Co 2p, (c) Fe 2p, (d) Os 4f, and (e) O 1s XPS spectra of CoFe LDH and Os/CoFe LDH‐x. (f) The ratio of Co^3+^: Co^2+^ and Fe^3+^: Fe^2+^ varies with the Os loading amount.

Standard nitrogen adsorption‐desorption experiments at 77 K were conducted to assess the specific surface area and porosity parameters of CoFe LDH and Os/CoFe LDH‐x. The specific surface area of Os/CoFe LDH‐x closely matched that of CoFe LDH. Furthermore, analysis of the nitrogen adsorption isotherms at 77 K revealed that both CoFe LDH and Os/CoFe LDH‐x displayed type IV characteristics (Figure S4). The pore size distribution (PSD) from the adsorption branches of the isotherms, showed that the peaks in their pore size distributions are predominantly centered in the range of 2–3 nm. In specific terms, Os/CoFe LDH‐40 exhibited a Brunauer–Emmett–Teller (BET) specific surface area of 63 m^2^ g^−1^, accompanied by a total pore volume of 0.278 cm^3^ g^−1^ (Table S3). The CoFe LDH and Os/CoFe LDH‐x architectures feature a hierarchical porous configuration, which integrates mesopores for an enlarged electrochemically active surface area with macropores for enhanced ion diffusion and efficient oxygen bubble detachment [29].

### 2.2 OER Performance

The OER performance was systematically evaluated using linear sweep voltammetry (LSV) in a 1.0 m KOH electrolyte (Figure 3a). Among all investigated catalysts, Os/CoFe LDH‐40 exhibited the lowest overpotential, requiring only 240 mV to achieve a current density of 10 mA cm^−2^. This performance significantly exceeds that of other catalysts, CoFe LDH (409 mV), Os/CoFe LDH‐5 (266 mV), Os/CoFe LDH‐20 (262 mV), Os/CoFe LDH‐60 (257 mV), Os/CoFe LDH‐80 (319 mV) and IrO_2_ (296 mV) (Figure 3b), indicating that the Os/CoFe LDH‐40 catalyst is optimal. The activity of Os/CoFe LDH‐40 surpass the activity of most reported catalysts (Figure S5 and Table S4). The superior OER activity of Os/CoFe LDH‐40 can be attributed to the strong interaction between Os species and CoFe LDH, which enhances intrinsic activity and provides abundant active sites. The Tafel slope for Os/CoFe LDH‐40 was determined to be 57.27 mV dec^−1^, significantly lower than those of other comparative catalysts (Figure 3c), suggesting favorable reaction kinetics and a more efficient OER pathway. The reduced Tafel slope indicates a modification in the rate‐determining step, likely facilitated by the optimized electronic structure and enhanced charge transfer capability [30, 31]. Electrochemical impedance spectroscopy (EIS) analysis was performed to probe the electrode kinetics during the OER process. The obtained Nyquist plots were fitted using the equivalent circuit, where *R*_s_ represents the solution resistance, CPE is the constant phase element accounting for the surface roughness of the LDH‐based materials, and *R*_ct_ is the charge transfer resistance. The fitting results showed that Os/CoFe LDH‐40 possessed the smallest *R*_ct_ (Figure 3d). The *R*_ct_ values followed the order: CoFe LDH > Os/CoFe LDH‐80 > Os/CoFe LDH‐5 > Os/CoFe LDH‐20 > Os/CoFe LDH‐60 > Os/CoFe LDH‐40. This trend confirmed a significantly improved interfacial charge transfer efficiency, facilitating faster reaction kinetics. Additionally, the *R*_s_ was about 4 Ω for all catalytic systems, including the optimal Os/CoFe LDH‐40. The narrow variation in *R*_s_ across all samples indicates that the ohmic drop arising from the electrolyte and cell configuration is consistent and does not contribute to the observed differences in catalytic activity. Therefore, the enhanced OER performance of Os/CoFe LDH‐40 can be attributed primarily to the improved interfacial charge transfer kinetics (as reflected by the significantly decreased *R*_ct_) rather than changes in solution resistance.

**FIGURE 3:**
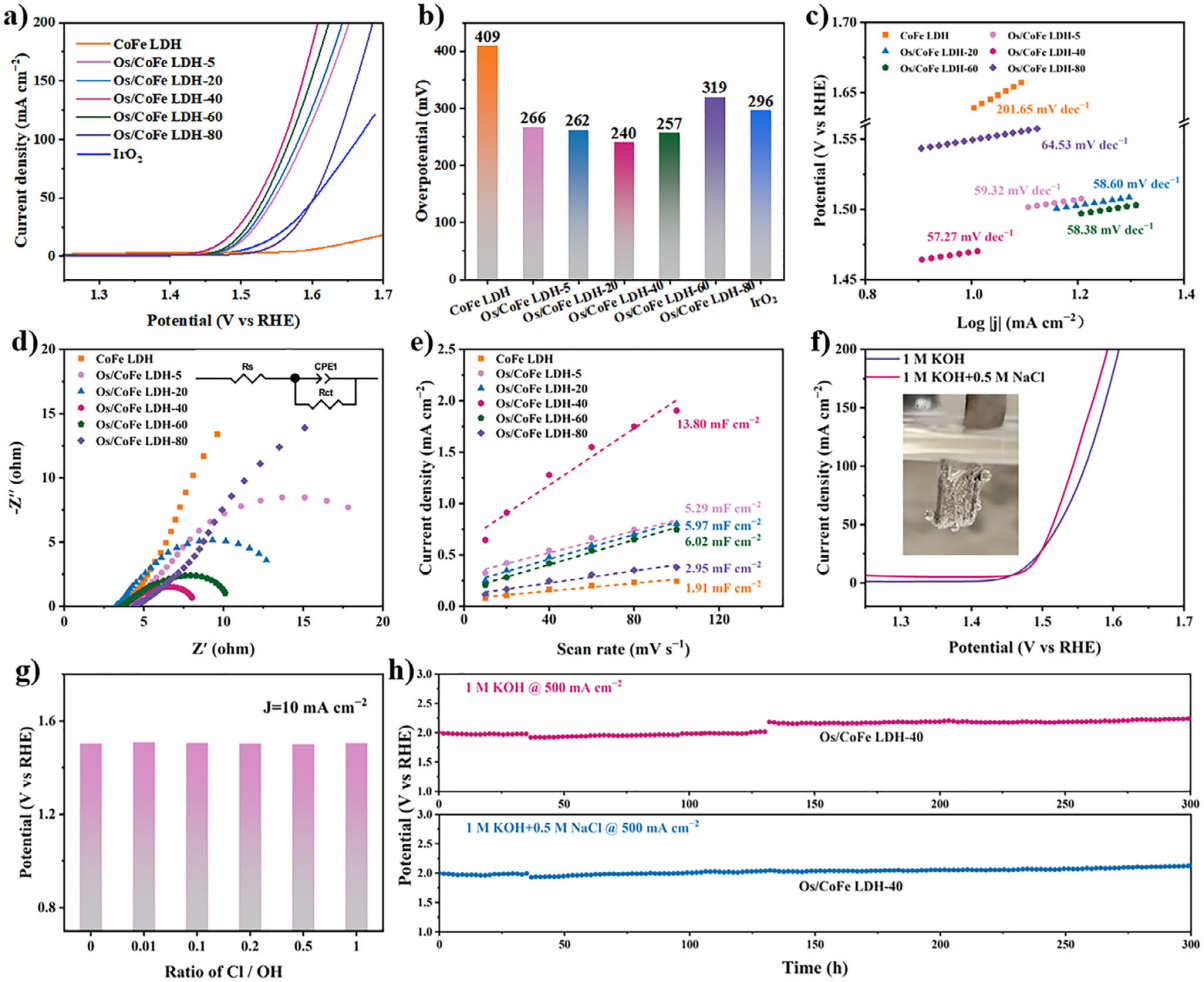
Electrocatalytic OER performance of CoFe LDH, Os/CoFe LDH‐x and IrO_2_. (a) LSV curves conducted in 1.0 m KOH. (b) Overpotential histogram at 10 mA cm^−2^ and (c) corresponding Tafel slopes. (d) Nyquist diagrams, inset shows the used equivalent circuit. (e) *C*_dl_ diagrams. (f) LSV curves of Os/CoFe LDH‐40 in 1.0 m KOH and simulated alkaline seawater, inset is the photograph of the work electrode during OER process. (g) Overpotential at 10 mA cm^−2^ in simulated alkaline seawater with different Cl^−^ concentrations. (h) Chronoamperometry curves of Os/CoFe LDH‐40 at 500 mA cm^−2^ in 1.0 m KOH and simulated alkaline seawater.

The electrochemical active surface area (ECSA) was assessed through CV measurements at various scan speeds in non‐Faradaic intervals (Figure S6), providing insight into the available active surface area. With the *C*_dl_ value of 13.80 mF cm^−2^ (Figure 3e), the ECSA value of Os/CoFe LDH‐40 calculated according to Equation (1) reaches 345.00 cm^2^, which is markedly higher than that of CoFe LDH (47.75 cm^2^), Os/CoFe LDH‐5 (132.25 cm^2^), Os/CoFe LDH‐20 (149.25 cm^2^), Os/CoFe LDH‐60 (150.50 cm^2^), and Os/CoFe LDH‐80 (73.75 cm^2^). The enhanced ECSA stems from the combined effects of the nanosheet morphology and the uniformly dispersed Os NPs. The roughness factor (RF = ECSA/geometric area) was further calculated to evaluate the intrinsic surface roughness of the catalysts. With a geometric electrode area of 0.25 cm^2^, the RF values of CoFe LDH, Os/CoFe LDH‐5, Os/CoFe LDH‐20, Os/CoFe LDH‐40, Os/CoFe LDH‐60, and Os/CoFe LDH‐80 were determined to be 191, 529, 597, 1380, 602, and 295, respectively. The RF value increases progressively with Os content, peaking at 1380 for Os/CoFe LDH‐40, which indicates that moderate Os loading effectively enhances surface roughness and exposes abundant accessible active sites. Beyond this optimal loading, however, the RF value declines markedly to 602 and 295 for Os/CoFe LDH‐60 and Os/CoFe LDH‐80, respectively, likely due to nanoparticle aggregation and/or pore blockage, which reduce the electrochemically active surface area.

The application of Os/CoFe LDH‐40 in seawater was also explored by comparing the catalyst's performance in 1.0 m KOH vs. simulated alkaline seawater (Figure 3f). Furthermore, the OER performance within varying the seawater concentration was investigated (Figure 3g), revealing that its overpotential remained relatively unchanged. This highlighted the robust performance of Os/CoFe LDH‐40 in resisting Cl^−^ corrosion within seawater environments. Accelerated degradation tests conducted via chronopotentiometry (CP) assessed the catalyst's long‐term operational stability. The potential increased by 11% after CoFe LDH operated in 1.0 m KOH less than 50 h (Figure S7). More impressively, after loading Os NPs, during 300 h of continuous operation at a current density of 500 mA cm^−2^ in both KOH and simulated alkaline seawater electrolyte, the catalyst maintained a stable potential response (Figure 3h), highlighting its exceptional durability. Post‐stability test characterizations of the catalyst structure and surface were performed to monitor dynamic surface changes during the oxygen evolution process, as understanding its reconstruction behavior is crucial for elucidating the true active phase and reaction mechanism. ICP‐MS results showed that after 300 h of stability testing in simulated alkaline seawater electrolyte, the leaching mass of Os is merely 1.53 µg. XRD and SEM results (Figures S8 and S9) confirmed that the nanosheet array structure of Os/CoFe LDH‐40 remained largely intact after prolonged electrolysis. Specifically, XRD patterns showed no significant changes in the crystalline phase or lattice parameters, indicating the preservation of the LDH structure. Furthermore, dispersed nanosheets and Os NPs were still observed in the TEM images, which confirmed that agglomeration did not occur (Figure S10a). According to the EDS mapping, the elements of Os, Co, Fe, and O in the sample were still uniformly distributed (Figure S10b). These results collectively indicate the significant structural resilience of the catalyst under extended operating conditions.

### 2.3 Investigation of Oxygen Evolution Mechanism

To elucidate the oxygen evolution mechanism at the molecular level, differential electrochemical mass spectrometry (DEMS) was employed. The real‐time monitoring recorded the mass spectrometry signals for ^32^O_2_, ^34^O_2_, and ^36^O_2_, allowing for the direct observation and differentiation of oxygen atoms originating from various sources, providing critical insights into the OER pathway. Initially, the catalyst was labeled with ^18^O by conducting CV for five cycles in a 1.0 m KOH solution prepared with H_2_^18^O. As shown in Figure 4a, Os/CoFe LDH‐40 exhibits a strong ^36^O_2_ signal accompanied by a weaker ^34^O_2_ signal, indicating the co‐existence of the adsorbate evolution mechanism (AEM) and the lattice oxygen mechanism (LOM). The AEM pathway involves O─O bond formation via coupling of adsorbed oxygen‐containing intermediates, while the LOM pathway involves direct participation of lattice oxygen atoms in O_2_ formation. Subsequently, the electrodes and electrolytic cell were rinsed with H_2_^16^O to remove physically adsorbed H_2_^18^O, then testing was performed in a H_2_^16^O‐based electrolyte. As shown in Figure 4b, the ^36^O_2_ signal nearly disappears, while a weak ^34^O_2_ signal persists, clearly indicating the participation of pre‐incorporated ^18^O from the catalyst lattice in the O─O bond formation process, consistent with LOM behavior. Based on the combined DEMS signals of ^34^O_2_ and ^36^O_2_ (Equation 2), the atomic ratio of lattice oxygen participation in Os/CoFe LDH‐40 (18a) was determined to be 0.16 (Figure S11). This value is significantly higher than the expected value from the pure AEM process (18a≈0.01), but significantly lower than the theoretical maximum of the completely random LOM process (18a≈0.5). The intermediate 18a value, combined with the simultaneous detection of ^34^O_2_ and ^36^O_2_ signals, clearly indicates that the OER on Os/CoFe LDH‐40 is carried out through a mixed mechanism involving both AEM and LOM pathways (Figure S12). This observation provides direct evidence for the involvement of lattice oxygen in the OER process, which may enhance reaction kinetics by offering an alternative pathway with a lower energy barrier.

**FIGURE 4:**
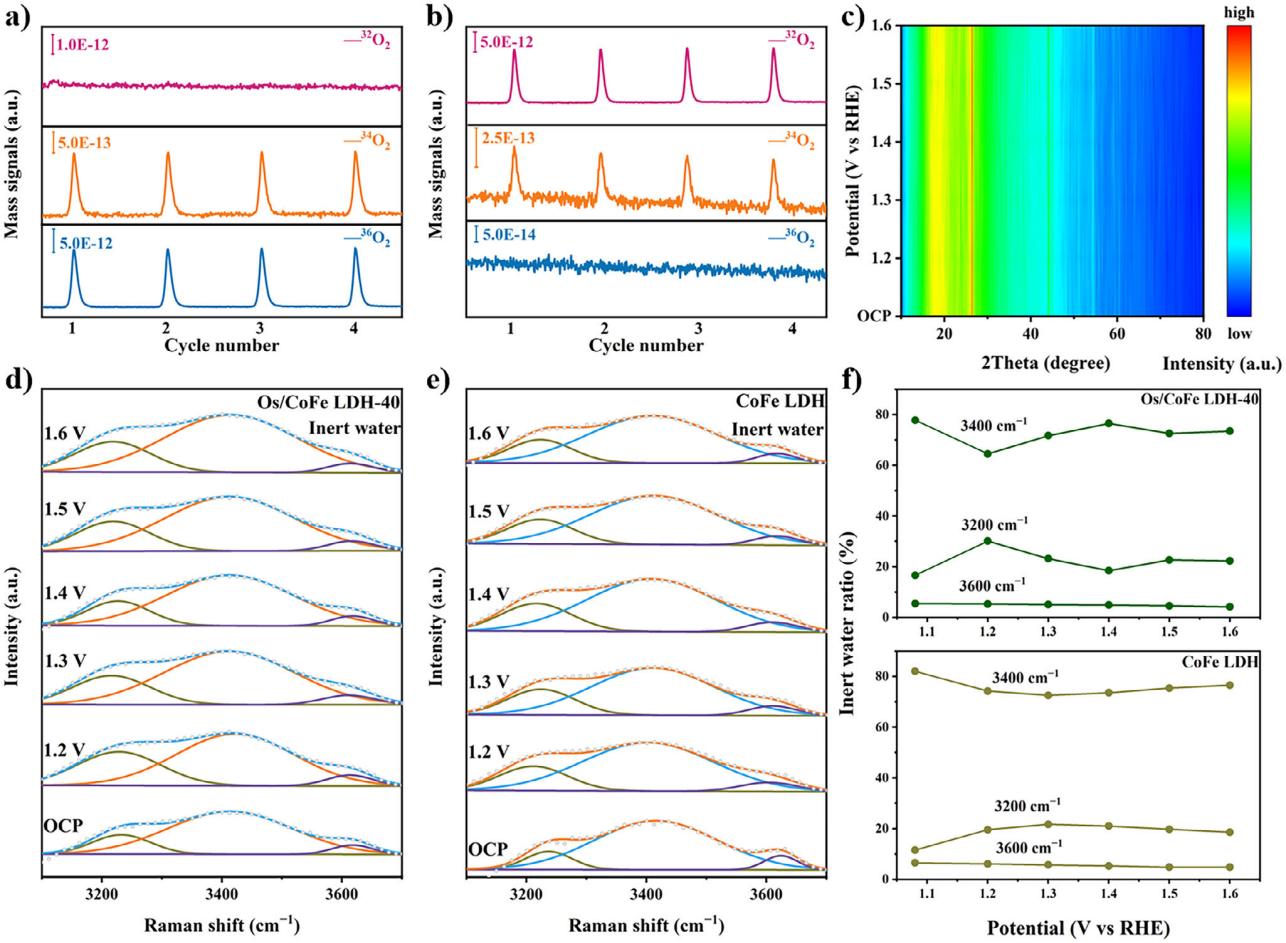
In situ characterization during the catalysis process. DEMS results in (a) 1.0 m KOH solution prepared with H_2_^18^O, (b) 1.0 m KOH solution prepared with H_2_^16^O for Os/CoFe LDH‐40. (c) XRD patterns were recorded under increasing applied potentials from OCP to 1.6 V vs. RHE without iR correction with Os/CoFe LDH‐40. In situ Raman spectra of (d) Os/CoFe LDH‐40 and (e) CoFe LDH at different applied potentials (OCP to 1.6 V vs. RHE) without iR correction. (f) The water structure evolution at the interface of Os/CoFe LDH‐40 and CoFe LDH from OCP to 1.6 V vs. RHE.

In situ XRD patterns (Figure 4c) showed that the main diffraction peaks of Os/CoFe LDH‐40 virtually no shift with the gradual application of potential, once again verifying its excellent structural stability. To better elucidate the electrocatalytic mechanism of Os/CoFe LDH‐40 during the OER process, in situ Raman spectroscopy measurements were conducted from the open‐circuit potential (OCP) to 1.60 V (vs. RHE) in a 1.0 m KOH solution for Os/CoFe LDH‐40 and CoFe LDH, respectively. In situ Raman enabled a deep investigation into how osmium influences the behavior of interfacial water. Deconvolution analysis of the broad peak corresponding to the O─H stretching vibration modes of interfacial water revealed three distinct water molecular structures (Figure 4d,e). The peak at 3200 cm^−1^ is attributed to O‐H stretching modes associated with the tetrahedral structure of bulk‐like water molecules. The characteristic peaks at 3400 and 3600 cm^−1^ correspond to hydrogen‐bonded O─H spanning the interface and non‐hydrogen‐bonded O─H oriented toward the surface, respectively. The water affinity of the catalyst is characterized by the ratio of O─H bonds bound to the surface. Consequently, the weaker vibrational peak of non‐hydrogen‐bonded H_2_O near 3600 cm^−1^ indicated relatively stronger hydrophilicity on the catalyst surface. Without applied potential, dispersion interactions dominated the adsorption capacity of interfacial water on the catalyst surface [32]. Upon the application of voltage, the proportion of surface inert water species decreased, signaling the formation of surface hydrogen bonds. To quantitatively investigate the influence of interfacial water on proton transfer, the potential‐dependent proportions of the three peaks were plotted in Figure 4f and Figure S13. As the voltage was applied, the proportion of inert water in Os/CoFe LDH‐40 was lower compared to that in CoFe LDH, implying that the Os/CoFe LDH‐40 surface was more hydrophilic, which was more favorable for the OER process.

### 2.4 Density Functional Theory Calculations

To elucidate the OER mechanisms in CoFe LDH and Os/CoFe LDH‐40, density functional theory (DFT) calculations were implemented to focus on CoFe LDH and Os/CoFe LDH crystal structure, based on modified Co hydroxide single‐crystal structures (Figure S14) [33]. Differential charge density, partial density of states (PDOS), and Gibbs free energies were analyzed for both CoFe LDH and Os/CoFe LDH models. The Os/CoFe LDH model exhibited significant charge redistribution around the Os site owing to supported‐metal‐particle heterostructures (Figure S15). To examine the impact of the electronic structure on the adsorption of OER intermediates [34], the corresponding PDOS of Co, Fe, and Os sites were calculated (Figure 5a,b). Note that the calculated d‐band centers for CoFe LDH and Os/CoFe LDH are −0.912 and −1.004 eV, respectively. With the introduction of Os, the leftward shift of the d‐band electrons decreases the chemical bond formation activity between the catalyst and the adsorbate, leading to an optimized adsorption energy for oxygen intermediates at the active sites [35]. This indicated that Os NPs loading significantly optimized the electronic structure of CoFe LDH, thereby enhancing its catalytic activity in the OER.

**FIGURE 5:**
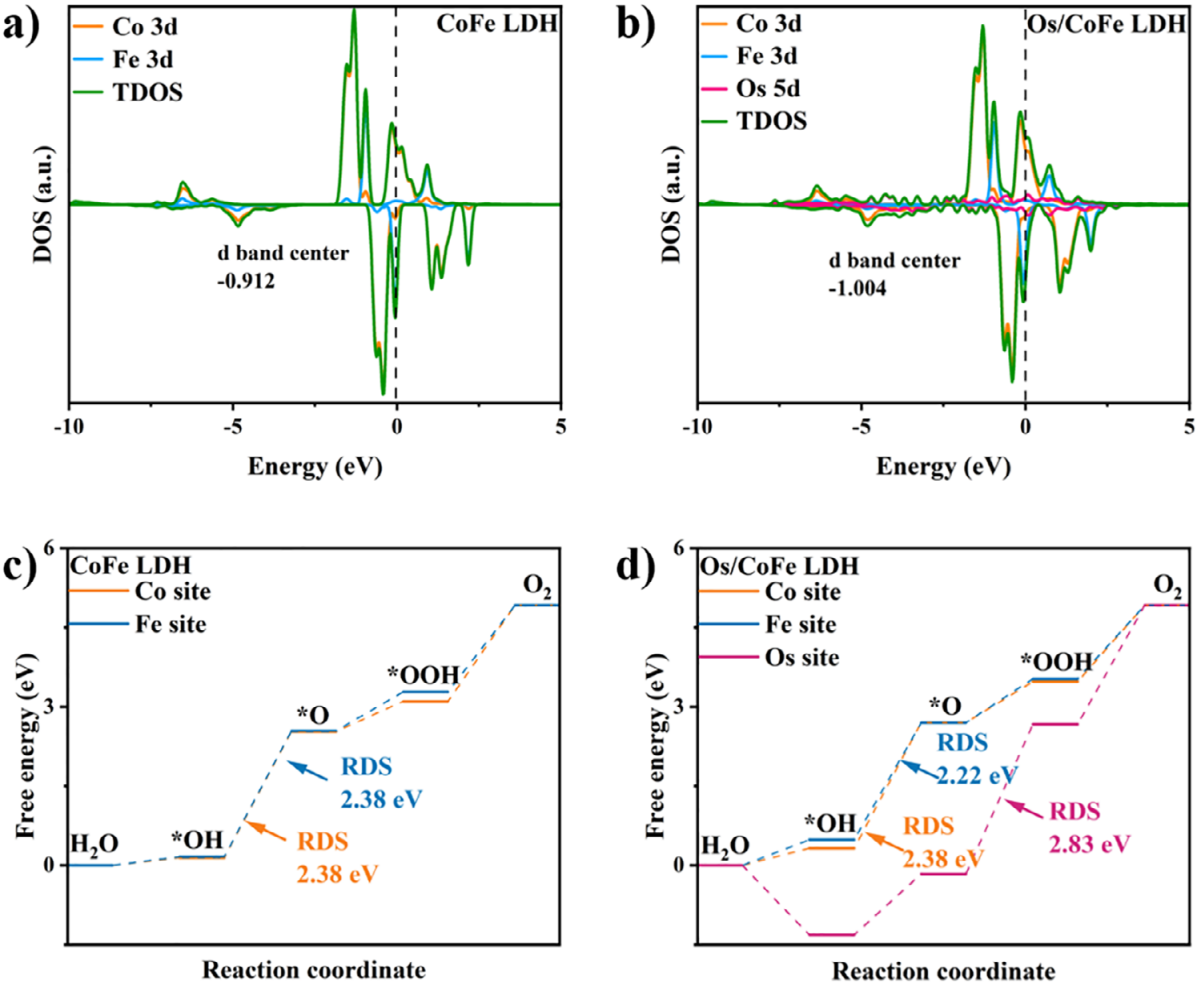
DFT calculations on the origin of enhanced OER mechanisms. DOS analysis of (a) CoFe LDH and (b) Os/CoFe LDH with dashed lines depicted the Fermi level (EF). (c) Gibbs free energy (ΔG) reaction diagrams for the Co and Fe sites of CoFe LDH at 0 V. (d) ΔG reaction diagrams for the Co, Fe, and Os sites of Os/CoFe LDH at 0 V.

To understand the OER process, the Gibbs free energies (ΔG) of adsorbed OER intermediates for the CoFe LDH and Os/CoFe LDH models were calculated (Figure 5c,d) [36]. In alkaline electrolytes, OER follows four steps [37]:*→*OH, *OH→*O, *O→*OOH and *OOH→* +^1^/_2_O_2_. As shown in Figures S16a and S17a, at the Co sites in the both models, the OER process was non‐spontaneous at 0 V. At 1.23 V, two steps (that is, *→*OH and *O→*OOH) were spontaneous, while two steps (that is,*OH→*O and *OOH→* +^1^/_2_O_2_) were not. At Fe sites, as for CoFe LDH, the rate‐determining step (RDS) was the formation of *O, where the ΔG was as large as 2.38 eV (U = 0 V, 1.15 eV at U = 1.23 V). This energy barrier of RDS for CoFe LDH was much larger than that for Os/CoFe LDH, which required only 2.22 eV (U = 0 V, 0.99 eV at U = 1.23 V) (Figures S16b and S17b). This explained why Os/CoFe LDH demonstrated a low overpotential and exceptional stability during alkaline OER. The lower computed energy barrier of RDS for Os/CoFe LDH relative to the one for CoFe LDH was consistent with the trend observed experimentally (Figure 3a). At Os sites (Figure S17c), only the first step (*→*OH) was calculated to be spontaneous (ΔG < O) at 0 V. At 1.23 V, Os/CoFe LDH exhibited two spontaneous (that is, *→*OH and*OH→*O) and two unfavourable (that is, *O→*OOH and *OOH→* +^1^/_2_O_2_) steps. Increasing the applied potential drove the transition of non‐spontaneous steps (*OH→*O) toward spontaneity.

To understand adsorption behavior, the thermodynamics of H_2_O adsorption over the CoFe LDH and Os/CoFe LDH were simulated, respectively. Both adsorption energy (ΔE_ads_) lie in a negative value, indicating that the adsorption process is spontaneous (Figure S18a,b). The Os/CoFe LDH (−0.35 eV) showed higher adsorption than that of CoFe LDH (−0.20 eV), illustrating that proper Os loading was beneficial to the adsorption of H_2_O. To further investigate the application of Os/CoFe LDH for OER in seawater, simulations of the Cl adsorption energy were also performed on both Os/CoFe LDH and CoFe LDH. The Os/CoFe LDH (−2.41 eV) showed weaker adsorption than that of CoFe LDH (−2.66 eV) (Figure S18c,d), illustrating that an appropriate Os loading is beneficial for reducing Cl adsorption, which explained the superior performance of Os/CoFe LDH in seawater OER.

### 2.5 AEM Water Electrolyzer Performance

The practical applicability of Os/CoFe LDH‐40 was evaluated in an AEMWE under simulated alkaline seawater conditions (1.0 m KOH + 0.5 m NaCl), representing a more realistic operating environment. As shown in Figure 6a, an Os/CoFe LDH‐40 anode combined with a commercial Pt/C cathode was used in an electrolyzer cell. This cell achieved a current density of 300 mA cm^−2^ at a cell voltage of 2.0 V at 25°C (Figure 6b). During long‐term stability assessment at 300 mA cm^−2^, it exhibited excellent durability (Figure 6c), highlighting its exceptional corrosion resistance and structural integrity in challenging seawater electrolytes. The comparative stability results of the AEM electrolyzer using seawater as the electrolyte (Figure S19 and Table S5) demonstrated that, compared with most other reported transition metal catalysts, Os/CoFe LDH‐40 exhibited superior durability. This outstanding device‐level performance strongly demonstrated that Os/CoFe LDH‐40 was a highly efficient and durable anode catalyst suitable for practical AEM water electrolysis applications, with the potential to enable cost‐effective hydrogen production from abundant seawater resources. In addition, compared with commonly used Ru‐based and Ir‐based OER catalysts, this study employs Os‐based catalysts, with the preparation cost of Os/CoFe LDH‐40 being only approximately 3.14 $ g^−1^, which provides new insights into the development of low‐cost and high‐performance seawater electrolysis (Table S6).

**FIGURE 6:**
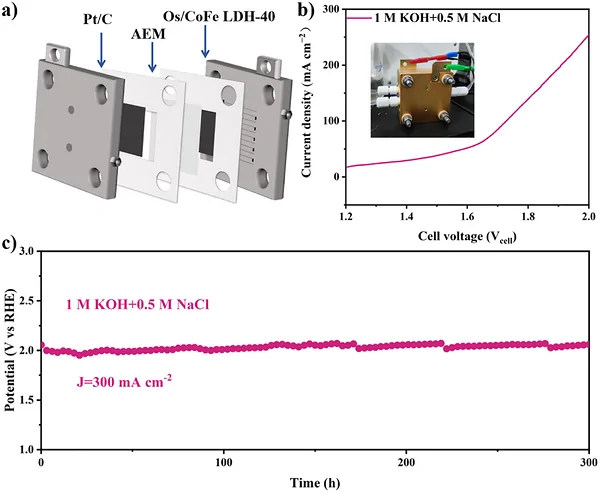
Performance of AEM seawater electrolysis with Os/CoFe LDH‐40. (a) Schematic diagram of the AEMWE electrolyzer components. (b) Polarization curve of Os/CoFe LDH‐40 in simulated alkaline seawater at room temperature, and inset is the photograph of the AEMWE experimental set‐up. (c) Durability test of Os/CoFe LDH‐40 at 300 mA cm^−2^ in an AEMWE system at room temperature.

## 3 Conclusion

In this study, the effective and durable electrocatalyst, Os/CoFe LDH‐40 was synthesized via a facile two‐step method comprising coprecipitation and impregnation. The anchored Os NPs induced strong interaction with the CoFe LDH host, facilitating charge transfer, stabilizing high‐valent Co, Fe species, and endowing the catalyst with excellent activity and durability for seawater OER. XPS analysis corroborated the retention of high‐valent Co and Fe states following Os loading. In situ Raman spectroscopy indicated that Os introduction optimizes the interfacial water structure. DFT calculations revealed that Os loading downshifted the d‐band center, optimized the adsorption of oxygen‐containing intermediates, and lowered the energy barrier of the RDS, thereby enhancing OER kinetics. Consequently, the catalyst delivered a current density of 10 mA cm^−2^ at overpotentials of 240 mV (1.0 m KOH) and 244 mV (simulated alkaline seawater), respectively. When Os/CoFe LDH‐40 was assembled into an AEMWE, it operated stably for over 300 h at 300 mA cm^−2^ under ambient conditions. This work demonstrates that Os NPs‐modified CoFe LDH represents a promising strategy for seawater‐based hydrogen production, offering a robust design paradigm for efficient and durable seawater OER catalysts and broadening the application horizon of Os‐based materials in electrocatalysis.

## 4 Experimental Section/Methods

### 4.1 Chemicals

Ferric nitrate nonahydrate (Fe(NO_3_)_3_·9H_2_O, 99.99%), cobalt(II) nitrate hexahydrate (Co(NO_3_)_2_·6H_2_O, 99.99%), sodium hydroxide (NaOH, ≥ 96%), sodium chloride (NaCl, ≥ 99.5%), sodium carbonate (Na_2_CO_3_, ≥ 99.5%) were purchased from Sinopharm Chemical Reagent Co., Ltd. Osmium trichloride hydrate (OsCl_3_·xH_2_O, 99.9%) was purchased from Shanghai Aladdin Biochemical Technology Co., Ltd. Iridium oxide (IrO_2_, 99.9%) was purchased from Suzhou Sinero Technology Co., Ltd. All the chemicals were used without further purification. Carbon fiber paper (Toray, TGP‐H‐060) was purchased from Suzhou Sinero Technology Co., Ltd. All aqueous solutions were prepared using deionized (DI) water (resistivity: 18.2 MΩ·cm).

### 4.2 Synthesis of CoFe LDH

CoFe LDH was synthesized by the co‐precipitation. Specifically, 40 mL solution A: 0.05 m Fe(NO_3_)_3_·9H_2_O and 0.1 m Co(NO_3_)_2_·6H_2_O, as well as 40 mL solution B: 0.75 m NaOH and 0.125 m Na_2_CO_3_ were first prepared. Then solutions A and B were simultaneously mixed to a beaker containing 40 mL DI water. Under magnetic stirring, adjust the pH value of the final suspension to 8.5 and monitor it with a pH meter at room temperature (25°C). The obtained suspension was aged at 25°C for 12 h. Subsequently, the precipitate was collected by centrifugation, washed with DI water and ethanol for each three times. The final precipitate was dried overnight in a vacuum oven at 60°C.

### 4.3 Synthesis of Os/CoFe LDH‐x

The 0.2 g as‐prepared CoFe LDH was added to 50 mL DI water and formed a colloidal suspension under magnetic stirring. Then, 10 mL freshly prepared aqueous OsCl_3_ solution (40 mg) at a concentration of 0.02 m NaOH was dropped into the above CoFe LDH colloidal suspension. The suspension was heated to 60°C while stirring and reacting for 12 h. Afterward, the precipitate was collected by centrifugation and washed three times with DI water and ethanol. Finally, the precipitate was dried overnight at 60°C in a vacuum oven, and the resulting product was named Os/CoFe LDH‐40. Samples with different Os contents loaded on CoFe LDH were prepared using the same method, but different amounts of OsCl_3_·xH_2_O precursors (5, 20, 60, and 80 mg) were used.

### 4.4 Material Characterization

The crystalline phase was collected through powder X‐ray diffraction (XRD, Bruker D8 Advance). The morphological information was observed via scanning electron microscope (SEM, Hitachi S4800) and transmission electron microscope (TEM, FEI Talos F200X) equipped with an energy‐dispersive X‐ray spectrometer (EDS). The chemical state of elements was determined by X‐ray photoelectron spectroscopy (XPS, Thermo Scientific K‐Alpha, USA) with a monochromatic Al Kα radiation source. The functional groups were identified by a Fourier transform infrared (FTIR) spectrometer (Thermo Scientific Nicolet iS10). The chemical composition of the samples was further evaluated by quantitative analysis using inductively coupled plasma‐mass spectrometry (ICP‐MS, Agilent 7800, USA). The Brunauer–Emmett–Teller (BET) specific surface area was collected through surface area and pore size analyzer (JW‐TB400, Beijing JWGB Instruments Co., Ltd.).

### 4.5 Electrochemical Measurement

The electrochemical performance was tested on an electrochemical workstation (CHI760E). All reactions were carried out in a standard three‐electrode system at room temperature. All electrochemical tests used a saturated Hg/HgO electrode as the reference electrode, a carbon rod as the counter electrode, and a carbon paper (CP) coated with catalyst as the working electrode. To prepare the catalyst slurry, 5 mg catalyst, 710 µL ethanol, 250 µL isopropanol, and 40 µL of 5 wt.% Nafion solution were mixed and ultrasonicated. Then as‐prepared slurry was applied onto carbon paper within 0.5 cm × 0.5 cm working area. In this study, all potentials were referenced to the reversible hydrogen electrode (RHE), and the calculation formula was *E*_RHE_ = *E*_Hg/HgO_ + 0.059 × *pH* + 0.098. To study the intrinsic activities of the catalysts, an iR compensation (85%) was used for the linear sweep voltammetry (LSV) data unless otherwise stated. The polarization data was collected at a scan rate of 5 mV s^−1^ in 1.0 m KOH electrolyte. Electrochemical impedance spectroscopy (EIS) measurements were performed over a frequency range of 100 kHz to 0.1 Hz, with an AC amplitude of 10 mV and an applied potential of 0.58 V vs. Hg/HgO. The ECSA was determined by the double layer capacitance (*C*_dl_), which was calculated based on the corresponding cyclic voltammetry (CV) curves, with scan rates of 10, 20, 40, 60, 80, and 100 mV s^−1^. The ECSA of the catalyst was estimated as follows:

(1) $$\mathrm{ECSA}=\frac{C_{\mathrm{dl}}}{C_{s}}$$

with the value of *C*_s_ (specific capacitance) being 0.04 mF cm^−2^.

The Tafel slope was calculated as follows:

η = a + blogi, where η is the overpotential, a is the Tafel constant, b is the Tafel slope, and i is the current density.

### 4.6 AEM Water Electrolyzer Performance

For the anion exchange membrane electrolysis (AEMWE) test, the anion exchange membrane was first immersed in 1.0 m KOH solution for 24 h for pre‐treatment, and then the membrane was stored in DI water for subsequent AEMWE evaluation. The newly synthesized Os/CoFe LDH‐40 and the commercial Pt/C (40%) electrodes were used as the anode and cathode catalysts, respectively. To prepare the catalyst ink, 10 mg of the catalyst was dispersed in 1 mL solution with a volume ratio of 3:1 of water and ethanol. Then, 40 µL of 5 wt.% Nafion solution was added to the mixture. The prepared catalyst ink was dropped onto a 1.0 cm × 1.0 cm carbon paper area to prepare the anode catalyst. Finally, the electrodes were used for the evaluation of catalytic performance in the AEMWE which was tested at room temperature, using a peristaltic pump to deliver 1.0 m KOH + 0.5 m NaCl electrolyte at a flow rate of 30 mL min^−1^. The long‐term stability test was conducted at a current density of 0.3 A cm^−2^. All AEMWE tests were measured without iR compensation.

### 4.7 In Situ Isotope‐Labeled DEMS Measurements

Isotope‐labeled differential electrochemical mass spectrometry (DEMS) measurements were carried out on the PM‐DEMS (Shanghai Pro‐tech Co., Ltd.). The Ag/AgCl and carbon rods were used as the reference electrode and counter electrode, respectively. The gold‐plated porous polytetrafluoroethylene (PTFE) membrane was used for the working electrode to support the sample, where the PTFE membrane was provided by Linglu Instrument Company, with a thickness of 60 µm, a pore diameter of 0.22 µm, and a porosity of 50%. First, in a 1.0 m KOH/H_2_^18^O electrolyte, 5 cycles of CV were performed on the Os/CoFe LDH‐40 catalyst at 20 mV s^−1^ over the potential window of 1.2–1.8 V (vs. RHE) to achieve oxygen isotope labeling on the catalyst surface. The gas products, including ^32^O_2_, ^34^O_2_, and ^36^O_2_, were detected in real time by mass spectroscopy. Subsequently, the electrodes and electrolytic cell were rinsed with H_2_^16^O to remove residual H_2_^18^O, and the same CV test was carried out in 1.0 m KOH/H_2_^16^O at 20 mV s^−1^ at the same potential window. The ^32^O_2_, ^34^O_2_, and ^36^O_2_ signal ratio were quantified by the ratio of signal peak area where the baseline was subtracted. The atomic fraction of ^18^O can be quantified by the following formula:

(2)

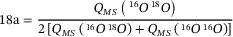

here, the Q refers to the integrated DEMS charge.

### 4.8 In Situ XRD Testing

In situ XRD measurements were carried out using a sealed in situ XRD electrochemical cell. Os/CoFe LDH‐40 coated on carbon paper, Ag/AgCl and carbon rod were used as the working electrode, reference electrode and counter electrode, respectively. The working electrode was prepared as described in the scheme in the electrochemical test section. The laser beam was focused onto the sample through the quartz window. Different voltages were applied to the electrodes at intervals of 100 mV, ranging from 1.2 to 1.6 V (vs. RHE).

### 4.9 In Situ Raman Spectra

In situ Raman testing was performed by a Raman spectrometer (LabRAM HR Evolution) with 532 nm excitation from an argon ion laser. Os/CoFe LDH‐40, Ag/AgCl, and carbon rod were employed as working electrode, reference electrode, and counter electrode, respectively. During OER measurement in 1.0 m KOH, the Raman spectra were collected at different potentials (100 mV intervals within the range from OCP to 1.6 V vs. RHE) by chronoamperometry test.

### 4.10 DFT Calculations

All theoretical calculations were performed using the projector augmented wave method and a plane‐wave basis set as implementation in the Vienna Ab Initio Simulation Package (VASP). The surface properties were optimized within GGA‐PBE. The Monkhorst‐Pack k‐point samplings were set as 3 × 2 × 1 for the geometry optimization, and 11 × 6 × 1 for the computation of electronic structure. The cutoff energy for plane‐wave basis functions was set to 500 eV with the energy change convergence criterion of 1 × 10^−5^ eV. Atomic positions were allowed to relax until the sum of the absolute forces reached down to 0.02 eV Å^−1^. The Gibb's free energies were calculated by:

(3) ΔG=ΔE+ΔZPE−TΔS

where the symbols represent the binding energy (Δ*E*), the change in zero‐point energy (ΔZPE), temperature (*T*), and the entropy change (Δ*S*) of the system, respectively.

## Author Contributions

**Xindong She**: methodology, data curation, writing – original draft, investigation. **Guosheng Zhou**: validation. **Kanghua Miao**: writing – review and editing. **Junjie Gong**: formal analysis. **Mingzhe Jiang**: visualization. **Xueting Zhu**: visualization. **Zhenzhen Wang**: writing – review and editing, conceptualization, supervision. **Shijie Shen**: project administration, funding acquisition. **Wenwu Zhong**: supervision, resources, funding acquisition.

## Conflicts of Interest

The authors declare no conflicts of interest.

## Supporting information




**Supporting File**: advs77998‐sup‐0001‐SuppMat.docx.

## Data Availability

The data that support the findings of this study are available from the corresponding author upon reasonable request.
