# Supplementary material for: Osmium Nanoparticle Anchoring on CoFe Layered Double Hydroxides Enables High‐Valent Species Stabilization and Efficient Seawater Oxygen Evolution
Source: Adv Sci (Weinh). 2026 Oct 14:e77998. Online ahead of print. doi: 10.1002/advs.77998 (PMC13668061; doi:10.1002/advs.77998)
Supplement: Supplementary file 1 [file ADVS-9999-e77998-s001.docx]

Supporting Information

**Osmium Nanoparticle Anchoring on CoFe Layered Double Hydroxides Enables High-Valent Species Stabilization and Efficient Seawater Oxygen Evolution**

*Xindong She^1^, Guosheng Zhou^1^, Kanghua Miao^2^, Junjie Gong^1^, Mingzhe Jiang^1^, Xueting Zhu^1^, Zhenzhen Wang*^, 1^, Shijie Shen*^, 1^, Wenwu Zhong*^, 2^*

*^1^* Zhejiang Key Laboratory for Island Green Energy and New Materials; School of Materials Science & Engineering, Taizhou University, Taizhou, Zhejiang 318000, P. R. China

^2^ Zhejiang Key Laboratory of Functional Ionic Membrane Materials and Technology for Hydrogen Production; School of Chemistry and Chemical Engineering, Shaoxing University, Shaoxing, Zhejiang 312000, P. R. China

Correspondence: Wenwu Zhong ([zhongww@tzc.edu.cn](mailto:zhongww@tzc.edu.cn,)); Shijie Shen (shensj@tzc.edu.cn); Zhenzhen Wang (zzwang@tzc.edu.cn)

**Figure S1.** SEM images of a-b) Os/CoFe LDH-5, c-d) Os/CoFe LDH-20, e-f) Os/CoFe LDH-60, and g-h) Os/CoFe LDH-80.

**Figure S2.** Fourier transform infrared spectroscopy of Os/CoFe LDH-40 and CoFe LDH.

**Figure S3.** XPS spectra of a) CoFe LDH, b) Os/CoFe LDH-5, c) Os/CoFe LDH-20, d) Os/CoFe LDH-40, e) Os/CoFe LDH-60, and f) Os/CoFe LDH-80.

**Figure S4.** N_2_ adsorption-desorption isotherms of a) CoFe LDH, b) Os/CoFe LDH-5, c) Os/CoFe LDH-20, d) Os/CoFe LDH-40, e) Os/CoFe LDH-60 and f) Os/CoFe LDH-80, and inset are their pore-size distributions.

**Figure S5.** Comparison of OER performance of Os/CoFe LDH-40 with other reported catalysts.

**Figure S6.** CV curves of a) CoFe LDH, b) Os/CoFe LDH-5, c) Os/CoFe LDH-20, d) Os/CoFe LDH-40, e) Os/CoFe LDH-60 and f) Os/CoFe LDH-80 at different scan rates in 1.0 M KOH.

**Figure S7.** Chronoamperometry curve of CoFe LDH at a current density of 300 mA cm^−2^ in 1.0 M KOH.

**Figure S8.** XRD pattern of Os/CoFe LDH-40 after a three-electrode system stability test conducted in a 1.0 M KOH solution.

**Figure S9.** SEM images of Os/CoFe LDH-40 after OER stability test at different magnifications.

**Figure S10.** a) TEM image of Os/CoFe LDH-40 after durability test. b) The corresponding elemental distribution maps of Os, Co, Fe, and O, respectively.

**Figure S11.** The proportion of ^18^O atoms from the lattice in the ^34^O_2_ and ^36^O_2_.

**Figure S12.** Adsorbate evolution mechanism (AEM) and lattice oxygen mechanism (LOM) of OER with Os/CoFe LDH.

**Figure S13.** The inert water ratio changes on the surface of CoFe LDH and Os/CoFe LDH-40 at different applied potential.

**Figure S14.** Atomic models of the CoFe LDH and Os/CoFe LDH.

**Figure S15.** The differential charge density of Os/CoFe LDH, where the yellow and green regions represent electronic accumulation and depletion, respectively.

**Figure S16.** Gibbs free energy diagrams on CoFe LDH at a) Co sites and b) Fe sites.

**Figure S17.** Gibbs free energy diagrams on Os/CoFe LDH at a) Co sites, b) Fe sites, and c) Os sites.

**Figure S18.** Comparison of adsorption energies of H_2_O and Cl. a) Adsorption energy of H_2_O by CoFe LDH. b) Adsorption energy of H_2_O by Os/CoFe LDH. c) Adsorption energy of Cl by CoFe LDH. d) Adsorption energy of Cl by Os/CoFe LDH.

**Figure S19.** Comparison of the stability performance for Os/CoFe LDH-40 with other reported catalysts for seawater electrolysis.

**Table S1.** The Os content in Os/CoFe LDH-40 was determined by ICP-MS analysis.

| Catalyst | Os (wt%) |
| --- | --- |
| Os/CoFe LDH-40 | 5.94% |

**Table S2.** The ratios of Co^3+^: Co^2+^ and Fe^3+^: Fe^2+^ in each catalyst from XPS results.

|  | Co^3+^:Co^2+^ | Fe^3+^:Fe^2+^ |
| --- | --- | --- |
| CoFe LDH | 1:1.3 | 1:1.1 |
| Os/CoFe LDH-5 | 1:1 | 1:1 |
| Os/CoFe LDH-20 | 1:0.7 | 1:1 |
| Os/CoFe LDH-40 | 1:0.7 | 1:1 |
| Os/CoFe LDH-60 | 1:0.7 | 1:1 |
| Os/CoFe LDH-80 | 1:0.7 | 1:1 |

**Table S3.** The physical properties of as-prepared samples.

| **Samples** | **S_BET_**  **(m^2^ g^−1^)** | **Pore volume (cm^3^ g^−1^)** | **Average pore size (nm)** | **Most frequent pore diameter (nm)** |
| --- | --- | --- | --- | --- |
| CoFe LDH | 65 | 0.366 | 22.3 | 2.7 |
| Os/CoFe LDH-5 | 51 | 0.282 | 21.8 | 2.6 |
| Os/CoFe LDH-20 | 58 | 0.268 | 18.4 | 2.4 |
| Os/CoFe LDH-40 | 63 | 0.278 | 17.6 | 2.9 |
| Os/CoFe LDH-60 | 106 | 0.441 | 16.6 | 2.1 |
| Os/CoFe LDH-80 | 137 | 0.458 | 13.3 | 2.1 |

**Table S4.** Comparison of the OER performance of Os/CoFe LDH-40 in 1.0 M KOH with previously reported advanced electrocatalysts.

| **Electrode materials** | **Overpotential (mV) @**  **j (mA cm^−2^)** | **Stability (h)** | **Reference** |
| --- | --- | --- | --- |
| **Os/CoFe LDH-40** | **240@10** | **300** | **This work** |
| PA0.5-CoFe-LDH/Co(OH)_2_ | 260@10 | 200 | [1] |
| a/c-CoFe-LDH | 269@10 | 100 | [2] |
| NiFe-V1.0/GCE | 254@10 | 240 | [3] |
| NiFe-LDH/NF-3.5 | 249@10 | 100 | [4] |
| CoMn-LDH-TiO_2_@C/NF-3 | 267@10 | 100 | [5] |
| NiFe-LDH/GA (1:1) | 257@10 | 56 | [6] |
| NiCu-LDH/NiTe@NF | 290@50 | 160 | [7] |
| NiCo-LDH/CN/AgPO | 289@10 | 50 | [8] |
| B-CD/NiFe LDH | 349@100 | 150 | [9] |
| Pt-NiSe@NiFe-LDH-Ov | 344@100 | 20 | [10] |
| 10% Sm@NiCu-LDH | 347@50 | 48 | [11] |

**Table S5.** The electrocatalytic performance of Os/CoFe LDH-40 was benchmarked against recently reported state-of-the-art electrocatalysts in 1 M KOH + 0.5 M NaCl electrolyte at 25 ℃.

| **Electrode materials** | **Cell voltage (V) @**  **j (mA cm^−2^)** | **Stability (h)** | **Reference** |
| --- | --- | --- | --- |
| **Os/CoFe LDH-40** | **2.0@300** | **300** | **This work** |
| NiFe LDH/Co(OH) | 1.9@1000 | 250 | [12] |
| NC-OX | 1.9@200 | 24 | [13] |
| SO_4_^2−^/CoFe LDH | 2.25@300 | 150 | [14] |
| NiFe-LDH/In(OH)_3_/NF | 2.009@200 | 200 | [15] |
| NZF-100 | 2.2@300 | 30 | [16] |
| NFCP-1 | 2.1@300 | 100 | [17] |
| NiFeCd-LDH/NF | 2.1@300 | 50 | [18] |
| Pt/WN@CPNiFe LDH/NF | 1.9@300 | 120 | [19] |
| NiFe-LDH/FeOOH | 2.1@500 | 100 | [20] |
| NiFeCo-LDH |  | 50 | [21] |

### **Table S6.** Estimated raw material costs for the synthesis of Os/CoFe LDH-40.

| **Precursor** | **Price ($ g^−1^)** | **Amount used (g)** | **Cost ($)** |
| --- | --- | --- | --- |
| Co(NO_3_)_2_·6H_2_O | 0.1736 | 0.808 | 0.14 |
| Fe(NO_3_)_3_·9H_2_O | 0.2361 | 1.160 | 0.27 |
| OsCl_3_·xH_2_O | 147.20 | 0.040 | 5.89 |

**References**

[1] Liu, S., Zhang, Y., Hao, L., et al. Organophosphorus layer‐modified CoFe‐LDH/Co(OH)_2_ electrocatalyst: Green synthesis and mechanistic insights into enhanced oxygen evolution activity, Small 2025, 21(49): e10412, <https://doi.org/10.1002/smll.202510412>.

[2] Gong, C., Li, W., Du, X., et al. Manipulating spin polarization by in-situ reconstructed amorphous/crystalline CoFe-LDH for efficient electrocatalytic water splitting, Nano Res. 2025, 18(9): 94907668, https://doi.org/[10.26599/NR.2025.94907668](https://doi.org/10.26599/NR.2025.94907668).

[3] Fang, X., Zhang R., Zeng X., et al. Electronic structure modulation of nickel-iron layered double hydroxide via vanadium doping for enhanced oxygen evolution reaction performance, RSC Adv. 2025, 15(51): 43312-43321, [https://doi.org/10.1039/D5RA03882K](https://doi.org/10.1039/D5RA03882K).

[4] Huang, Y., Liu, X., Li, X., et al. Interfacial oxidation using potassium ferrate to fabricate self-supported hydrophilic NiFe-LDH nanoarrays for overall water splitting at high current density, Sustainable Mater. Technol. 2022, 34: e00508, https://doi.org/10.1016/j.susmat.2022.[e00508](https://doi.org/10.1016/j.susmat.2022.e00508).

[5] Chu, X., He, Y., Wang, Y., et al. Electronic modulation of CoMn-LDH on TiO_2_@C via heterogeneous interface engineering to achieve high-performance overall water splitting, J. Alloys Compd. 2025, 1035: 181473, [https://doi.org/10.1016/j.jallcom.2025.181473](https://doi.org/10.1016/j.jallcom.2025.181473).

[6] Zhang, J., Cao, Q., Yu, X., et al. Interface synergistic effect of NiFe-LDH/3D GA composites on efficient electrocatalytic water oxidation, Nanomaterials 2024, 14(20): 1661, https://doi.org/[10.3390/nano14201661](https://doi.org/10.3390/nano14201661).

[7] Zheng, Y., Sun, Y., Wang, Q., et al. Interfacial engineering of crystalline-amorphous NiCu-LDH/NiTe@NF: Opening a synergistic pathway for superior oxygen evolution catalysis, J Alloys Compd. 2026, 1051: 186062, [https://doi.org/10.1016/j.jallcom.2026.186062](https://doi.org/10.1016/j.jallcom.2026.186062).

[8] Mandari, K., Im, Y., Kang, M. NiCo-LDH sheets and Ag_3_PO_4_ nanoparticles decorated on graphitic carbon nitride for efficient oxygen evolution reaction, J. Alloys Compd. 2024, 1004: 175860, [https://doi.org/10.1016/j.jallcom.2024.175860](https://doi.org/10.1016/j.jallcom.2024.175860).

[9] Wang, Y., Zhao, Z., Li, W., et al. Pre-introduced carbon dots on anode prior to nickel-iron layered double hydroxide growth for stable seawater splitting, Int. J. Hydrogen Energy 2026, 204: 153269, [https://doi.org/10.1016/j.ijhydene.2025.153269](https://doi.org/10.1016/j.ijhydene.2025.153269).

[10] Li, S., Guo, Y., Wang, Z., et al. A core–shell Pt–NiSe@NiFe-LDH heterostructure for bifunctional alkaline water splitting, Molecules 2025, 30(23): 4654, https://doi.org/10.3390/[molecules30234654](https://doi.org/10.3390/molecules30234654).

[11] Roy, S., Karmakar, A., Madhu, R., et al. Three-dimensional Sm-doped NiCu-LDH on Ni foam as a highly robust bifunctional electrocatalyst for total water splitting, ACS Appl. Energy Mater. 2023, 6(17): 8818-8829, [https://doi.org/10.1021/acsaem.3c01338](https://doi.org/10.1021/acsaem.3c01338).

[12] Zhang, W., Li, Z., Jang, H., et al. Synergistic chloride repulsion in hierarchical NiFe LDH/Co(OH)_2_ heterostructure enables industrial-grade current density and ultra-stability for seawater electrolysis over 4600 h, ACS Sustainable Chem Eng. 2026, 14: 5030-5042, https://doi.org/10.1021/[acssuschemeng.5c13313](https://doi.org/10.1021/acssuschemeng.5c13313).

[13] Pal, S., Sinha, N., Das, C., et al. Enhanced electrocatalytic performances of NiCr layered double hydroxides by oxalate intercalation in anion exchange membrane water electrolysis, ACS Appl. Mater. Interfaces 2025, 17(26): 37863-37878, [https://doi.org/10.1021/acsami.5c04189](https://doi.org/10.1021/acsami.5c04189).

[14] Yu, Y., Zhou, W., Zhou, X., et al. Taking advantage of activation potential coincidence to unlock stable direct seawater splitting, Adv. Funct. Mater. 2025, 35(20): 2419871, https://doi.org/[10.1002/adfm.202419871](https://doi.org/10.1002/adfm.202419871).

[15] Roy, S., Minhas, H., Lakshminarayanan, P., et al. Vertically grown face‐to‐face nano‐assembly of In(OH)_3_ and NiFe‐LDH enables interfacial charge separation for enhanced alkaline and seawater electrolysis, Adv. Funct. Mater. 2026, 36(24): e24906, https://doi.org/[10.1002/adfm.202524906](https://doi.org/10.1002/adfm.202524906).

[16]	Das, C., Sinha, N., Nair, A., et al. Chlorophobic iron hydrogen phosphite as OER‐active electrocatalyst in anion exchange membrane (sea) water electrolysis, Small 2025, 21(34): 2505781, <https://doi.org/10.1002/adfm.202524906>.

[17] Yuan, J., Zhou, W., Yu, Y., et al. Balanced anion engineering unlocks highly active and corrosion-resistant seawater splitting, Nano Energy 2026, 150: 111792, https://doi.org/10.1016/[j.nanoen.2026.111792](https://doi.org/10.1016/j.nanoen.2026.111792).

[18] Zhou, M., Wang, Y., Chen, Q. Engineering cadmium-doped NiFe-LDH nanosheet arrays via low-temperature etching for robust high-current-density seawater electrolysis, J. Mater. Chem. A 2025, 13(34): 28624-28634, [https://doi.org/10.1039/D5TA03887A](https://doi.org/10.1039/D5TA03887A).

[19] Geng, S., Chen, L., Wu, Y., et al. Lattice-matched metal/WN catalyst with highly oxygenophilic W sites for hydrogen production in seawater electrolyzer, J. Energy Chem. 2025, 105: 302-311, https://doi.org/[10.1016/j.jechem.2024.12.071](https://doi.org/10.1016/j.jechem.2024.12.071).

[20] Chen, Z., Chen, L., Yin, S., et al. Ultrafast in situ-formed FeOOH ultrathin overlayers on NiFe-LDH surfaces enable highly durable alkaline seawater oxidation, Inorg. Chem. Front. 2026, 13: 3541-3552, https://doi.org/10.1039/D5QI02094H.

[21] Park, Y., Jeong, J., Jang, M., et al. Ternary layered double hydroxide oxygen evolution reaction electrocatalyst for anion exchange membrane alkaline seawater electrolysis, J. Energy Chem. 2022, 75: 127-134, https://doi.org/[10.1016/j.jechem.2022.08.011](https://doi.org/10.1016/j.jechem.2022.08.011).
